# Challenging Ureteroscopy in Complex Anatomy: A Case Report

**DOI:** 10.7759/cureus.107466

**Published:** 2026-04-21

**Authors:** Santosh Patil, Siddanagouda Patil, Vinay S Kundargi, Anupam Banerjee

**Affiliations:** 1 Urology, Bijapur Lingayat District Educational (BLDE) Association (Deemed to be University), Shri B. M. Patil Medical College, Hospital and Research Centre, Vijayapura, IND

**Keywords:** anatomical variation, case report, difficult ureter, endourology, impacted ureteric calculus, ureteroscopy

## Abstract

Ureteroscopy is a well-established minimally invasive modality for the management of ureteric calculi; however, technical difficulty may arise in the presence of impacted stones and distorted urinary tract anatomy. We present a case of challenging ureteroscopic lithotripsy in a 24-year-old man with a symptomatic impacted distal ureteric calculus measuring approximately 9 × 8 × 10 mm, associated with hydroureteronephrosis and markedly altered pelvic orientation due to kyphoscoliosis. The patient also had underlying metabolic bone disease with renal osteodystrophy, which further complicated perioperative positioning and anesthetic management. Preoperative imaging demonstrated distorted pelvic anatomy, an abnormal ureteral course, and a high-density distal ureteric stone. During surgery, spinal deformity and pelvic tilt created a highly angulated ureteral path, reducing ureteroscopic maneuverability and increasing resistance to advancement. The stone was fragmented using a combined pneumatic and laser lithotripsy approach, and a double-J ureteral stent was placed to facilitate drainage and ureteral healing. The postoperative course was uneventful, with complete stone clearance and preservation of renal function. This case highlights the importance of recognizing anatomical distortion, skeletal comorbidity, and stone-related factors as causes of difficult ureteroscopy and underscores the value of careful preoperative imaging, individualized planning, and intraoperative adaptability in complex endourological cases.

## Introduction

Recent progress in endoscopic technology, improved optical resolution, and the availability of effective intracorporeal lithotripsy systems have made ureteroscopy one of the mainstays in the modern management of ureteral and renal calculi. The widespread application of semirigid and flexible ureteroscopes has made it possible to achieve high stone-clearance rates with lower morbidity, and most ureteric stones can now be treated by minimally invasive methods. However, despite advances in technology, ureteroscopy may still pose significant procedural difficulty in selected clinical situations. A challenging ureter during ureteroscopy is usually encountered in the presence of impacted calculi, mucosal edema, inflammatory changes, or anatomical variations. Impacted ureteric stones, especially those located in the lower ureter, are associated with prolonged operative time, increased resistance to scope advancement or stone retrieval, and a higher risk of ureteral injury. Recent literature highlights that difficulty during ureteroscopy is not uncommon and requires careful intraoperative judgment to prevent complications such as ureteral perforation, avulsion, or incomplete stone clearance [1].

Multiple patient- and stone-specific factors have been identified as predictors of ureteral difficulty during retrograde ureteroscopic lithotripsy. Stone size, duration of impaction, distal location, and associated hydronephrosis contribute to narrowing of the ureteral lumen and increased ureteral wall rigidity. Inflammatory edema and reduced ureteral compliance further complicate endoscopic navigation, often necessitating modification of operative strategy or staged intervention [2]. Although refinements in ureteroscope design, including the availability of single-use flexible ureteroscopes, have improved maneuverability and reduced instrument-related limitations, technical challenges continue to persist in anatomically complex cases [3].

Numerous techniques have been described to address a difficult ureter, including balloon dilation, preoperative stenting, and alternative endoscopic approaches. Balloon dilation can facilitate ureteral access in selected patients when performed cautiously, while pre-stenting may allow gradual ureteral accommodation before definitive stone management [4]. In cases where endoscopic progression remains unsafe or unsuccessful, reconstructive or laparoscopic interventions may be required, particularly in the presence of ureteral strictures or post-surgical anatomical distortion [5].

Anatomical variations and associated conditions such as hydronephrosis, spinal deformities, or an aberrant ureteral course further increase procedural complexity by altering the normal alignment of the urinary tract and affecting endoscopic ergonomics. These variables can markedly affect scope trajectory, instrument maneuverability, and access to the target calculus, emphasizing the need for individualized procedural planning and intraoperative adaptability [6,7].

This case report describes a technically challenging ureteroscopic procedure in a young male patient with an impacted distal ureteric calculus complicated by abnormal anatomical orientation. The report focuses on the intraoperative difficulties encountered, the significance of radiographic findings in demonstrating the altered ureteral trajectory, and the strategic decisions that enabled successful management despite unfavorable anatomical conditions.

## Case presentation

**Figure 1:**
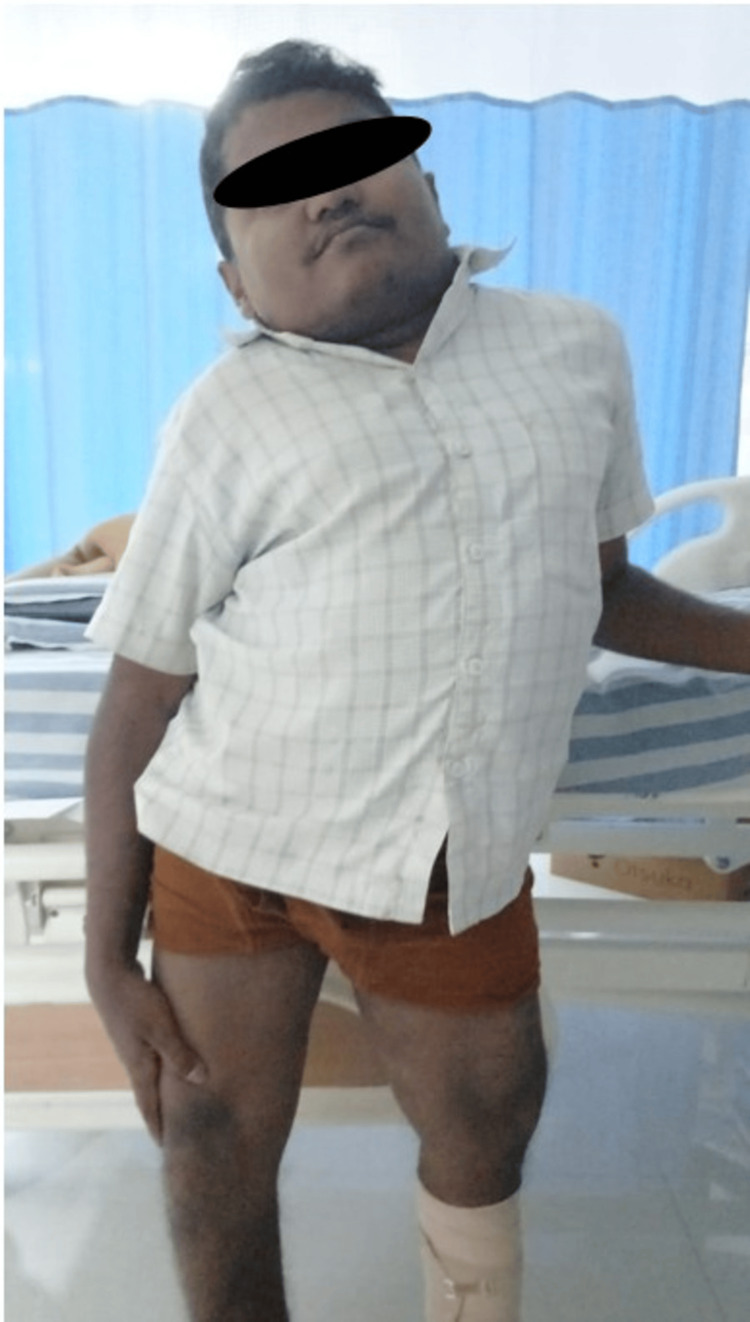
Clinical photograph demonstrating abnormal postural alignment. Clinical photograph of the patient demonstrating marked postural asymmetry with lateral deviation of the trunk. This abnormal posture reflects the underlying skeletal deformity and contributed to altered pelvic orientation, posing challenges in patient positioning and endoscopic ergonomics during ureteroscopy.

**Figure 2:**
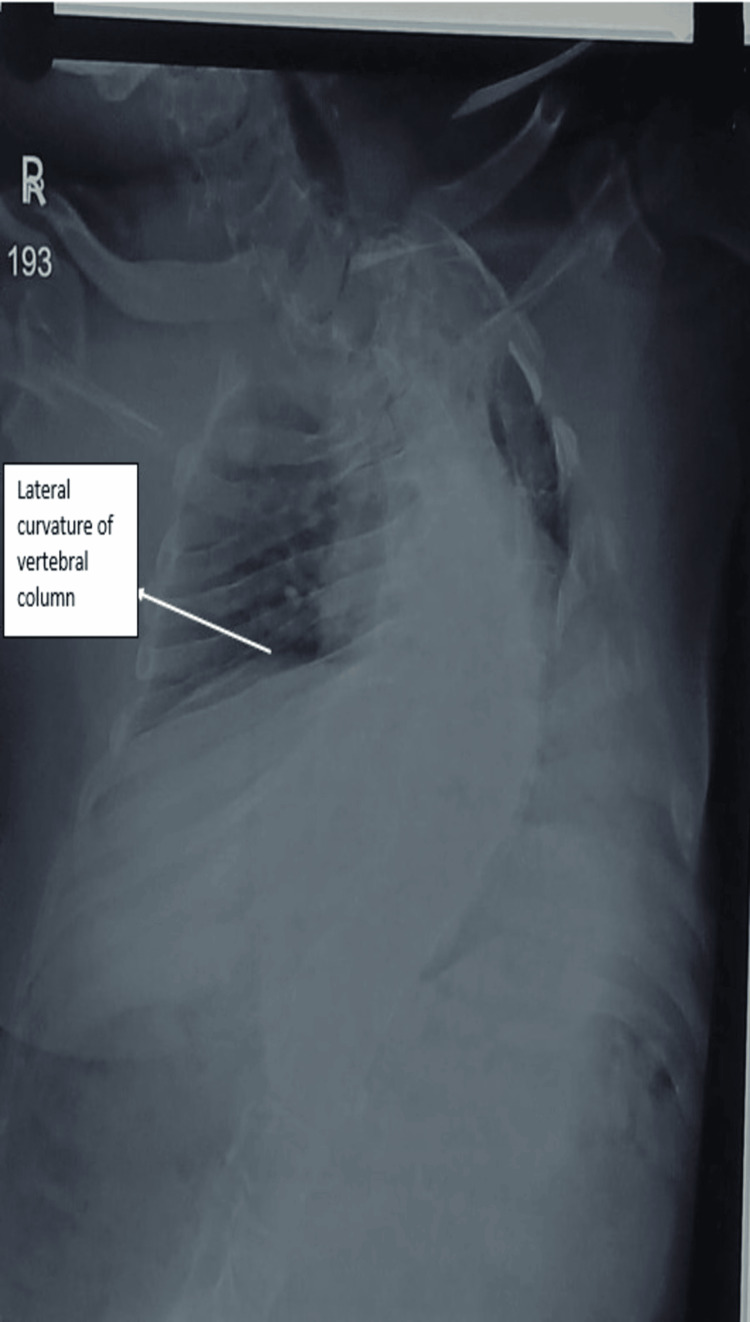
Chest radiograph showing abnormal curvature of the thoracic spine consistent with kyphoscoliosis. The white arrow indicates the lateral curvature of the vertebral column. This spinal deformity contributed to altered body alignment and indirectly influenced pelvic orientation and ureteral course during ureteroscopic intervention.

A 24-year-old male presented with bilateral flank pain for three months, with worsening symptoms over the preceding 15 days. There was no history of fever, dysuria, hematuria, or lower urinary tract symptoms. Written informed consent was obtained from the patient for publication of this case report and accompanying images. Clinical examination revealed abnormal postural alignment with lateral trunk deviation (Figure 1). Chest radiography confirmed thoracic spinal curvature consistent with kyphoscoliosis, establishing an underlying skeletal deformity that influenced patient positioning and pelvic orientation (Figure 2).

Hematological parameters, renal function tests, serum electrolytes, coagulation profile, and urine culture were all within normal limits, and the patient was considered fit for surgical intervention.

**Figure 3:**
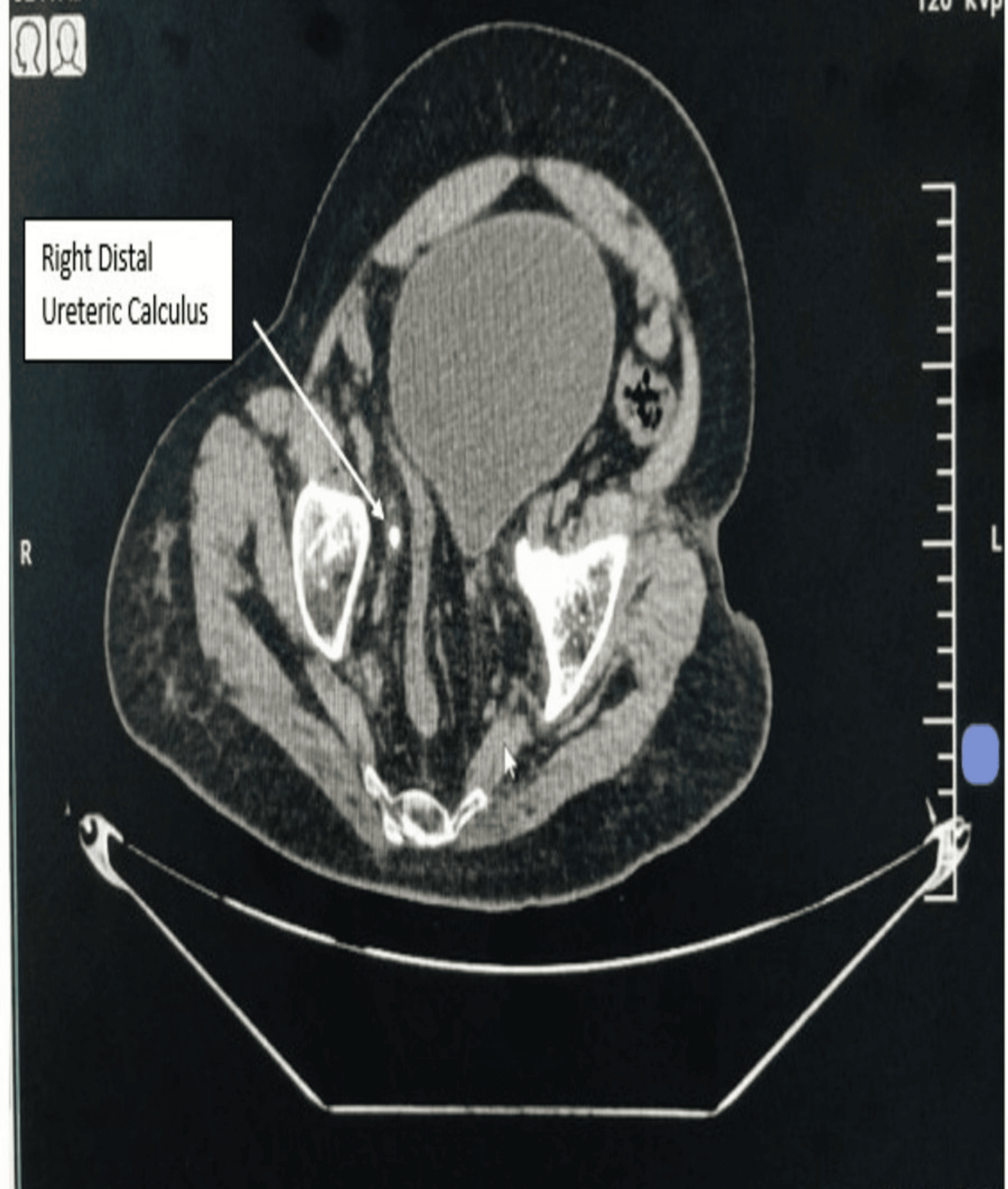
CT KUB axial section demonstrating distorted pelvic anatomy. Non-contrast CT of the kidneys, ureters, and bladder, axial section, demonstrating distorted pelvic anatomy with altered spatial relationships between the urinary bladder and surrounding structures. The white arrow indicates the right distal ureteric calculus. These findings contributed to difficulty in ureteroscopic navigation and access to the distal ureter. CT KUB: Computed Tomography of the Kidneys, Ureters, and Bladder.

**Figure 4:**
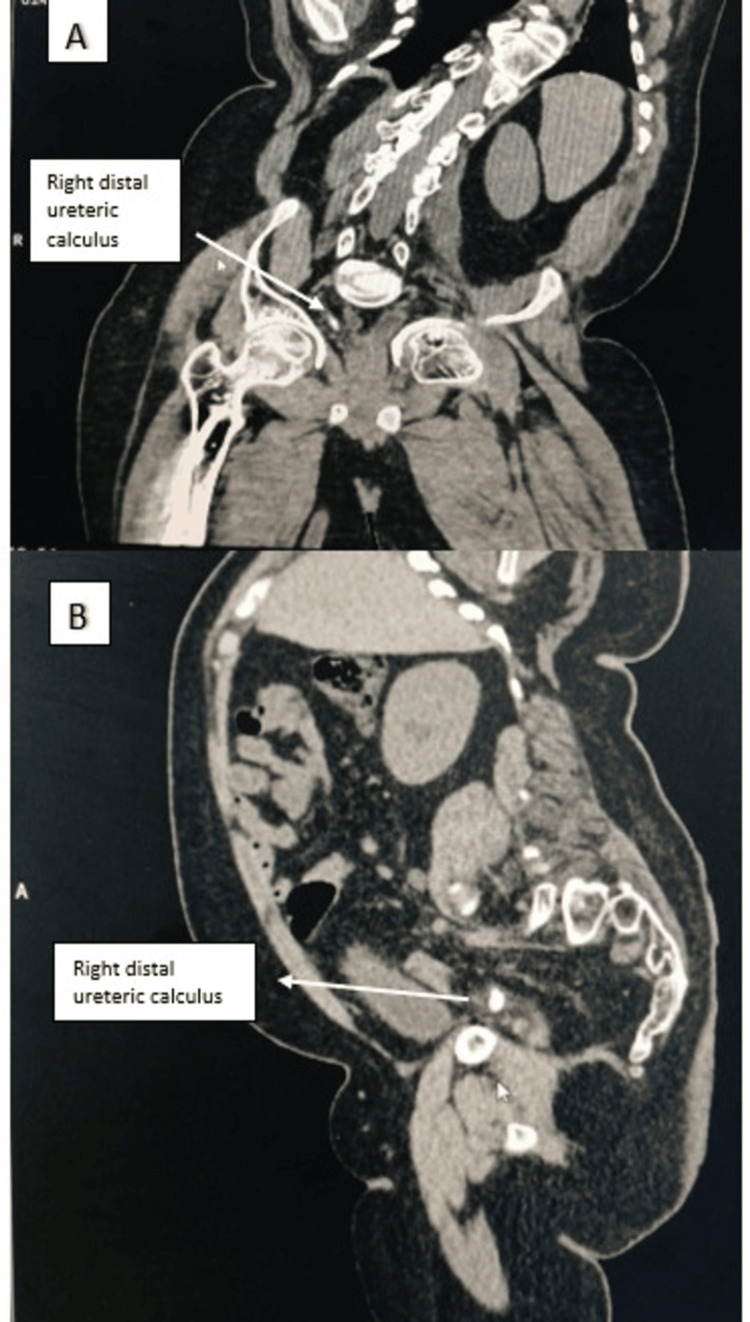
CT KUB images demonstrating an impacted distal ureteric calculus. CT images (coronal and sagittal) showing a right distal ureteric calculus indicated by the white arrow. (A) Coronal reformatted non-contrast computed tomography image demonstrating an impacted distal ureteric calculus with surrounding soft tissue changes suggestive of inflammatory edema. (B) Sagittal reformatted image showing the distal ureteric calculus with associated upstream ureteral dilatation, highlighting the altered ureteral course and increased resistance encountered during ureteroscopic advancement. CT KUB: Computed Tomography of the Kidneys, Ureters, and Bladder.

USG demonstrated mild right-sided hydroureteronephrosis. Preoperative non-contrast CT KUB was particularly valuable in this case because it delineated the impacted distal ureteric calculus, quantified stone density, demonstrated upstream ureteral dilatation, and, most importantly, revealed distorted pelvic anatomy with an altered ureteral course. These findings anticipated the likelihood of difficult ureteroscopic access and allowed the operating team to proceed with heightened caution and readiness to modify the endoscopic strategy if required. Non-contrast CT of the kidneys, ureters, and bladder revealed distorted pelvic anatomy with altered spatial orientation of the urinary bladder and distal ureter (Figure 3). A hyperdense impacted calculus measuring approximately 9 × 8 × 10 mm was identified in the distal right ureter, located approximately 12 cm proximal to the vesicoureteric junction, with associated upstream ureteral dilatation (Figures 4A-4B). The measured stone density of approximately 1250 Hounsfield units suggested a hard calculus with a high likelihood of fragmentation resistance.

Right-sided ureteroscopic lithotripsy was planned. Under spinal anesthesia, the patient was placed in the lithotomy position with careful padding and gentle handling because of underlying metabolic bone disease and renal osteodystrophy. Following cystoscopy, the right ureteric orifice was identified, and a safety guidewire was placed under direct vision. A semirigid ureteroscope was then advanced carefully into the distal ureter. Progress was difficult because of acute ureteral angulation, mucosal edema, and narrowing around the impacted calculus. Gentle torque control, minimal advancement force, and repeated realignment were required to avoid ureteral trauma. Fragmentation was performed using pneumatic lithotripsy followed by laser lithotripsy to achieve controlled disintegration of the dense impacted stone while minimizing retropulsion. Stone fragments were retrieved with grasping forceps, and the distal ureter was reinspected for residual fragments and mucosal injury. Because of ureteral edema and prolonged instrumentation, a 5 Fr, 26 cm double-J ureteral stent was placed at the end of the procedure.

The total operative time was approximately 2 hours and 30 minutes. Ureteral edema was assessed intraoperatively and graded as mild. Fluoroscopic guidance (C-arm) was not utilized, as the calculus was in the distal ureter and direct endoscopic visualization was sufficient for safe navigation.

Although flexible ureteroscopy was considered as an alternative strategy in view of the distorted ureteral course, the stone was in the distal ureter and could ultimately be accessed using a semirigid ureteroscope with cautious manipulation. Therefore, conversion to flexible ureteroscopy was kept as a backup option but was not required intraoperatively.

**Figure 5:**
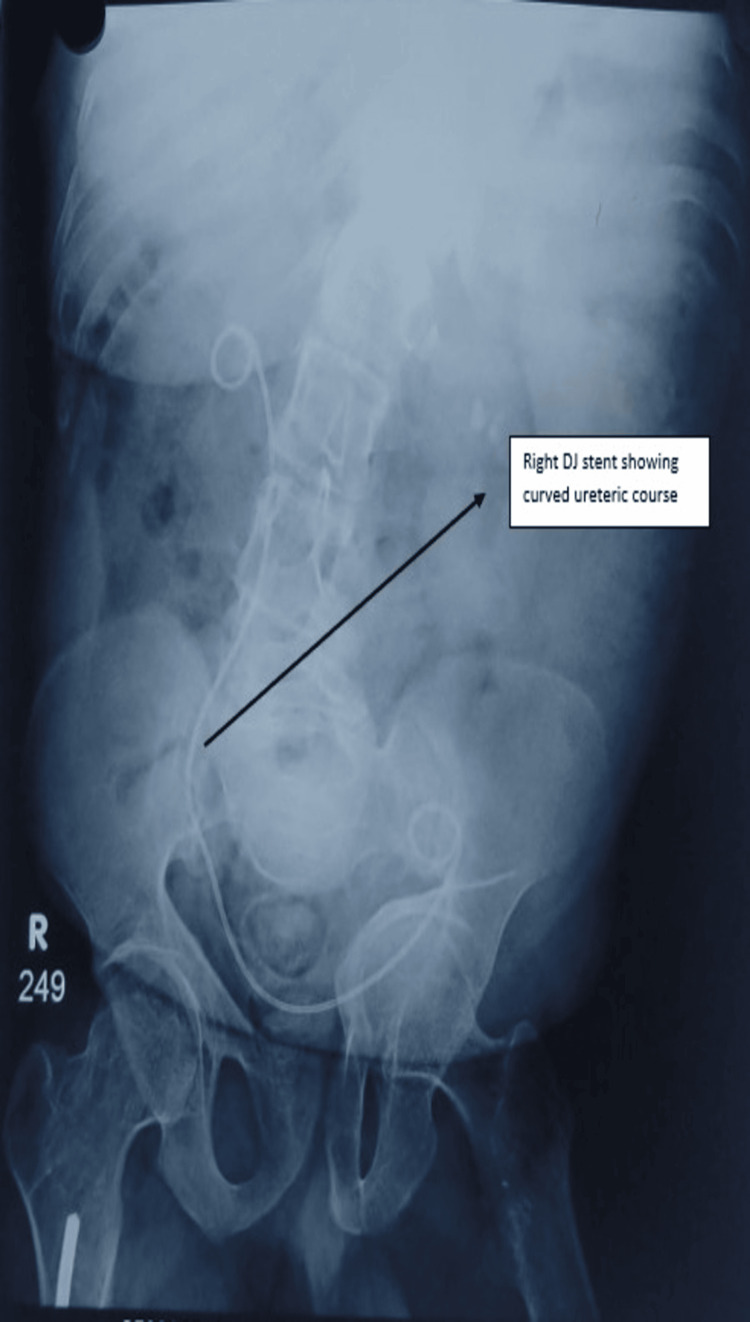
Post-procedure radiograph demonstrating double-J stent placement with marked ureteral angulation. Postoperative X-ray KUB showing stone clearance and the curved course of the right ureter. The black arrow indicates the course of the double-J stent. Plain radiograph obtained after ureteroscopy showing double-J stent placement with a nonlinear and markedly angulated ureteral course. The abnormal stent trajectory visually demonstrates the distorted ureteral anatomy encountered intraoperatively and highlights the technical difficulty involved in accessing and managing the distal ureteric calculus. KUB: Kidneys, Ureters, and Bladder.

Post-procedure radiography demonstrated the double-J stent in situ with a markedly angulated and nonlinear ureteral trajectory, visually reflecting the distorted anatomy encountered intraoperatively (Figure 5). The stent configuration corroborated the technical difficulty experienced during ureteroscopic access and manipulation. Stone fragments were subjected to analysis, which confirmed calcium oxalate monohydrate as the predominant component, with a minor proportion of calcium oxalate dihydrate. Stone culture was sterile, indicating no evidence of infective stone pathology. This was consistent with the high stone density observed on preoperative imaging and the resistance encountered during fragmentation. The postoperative course was uneventful. The patient remained hemodynamically stable and reported significant relief from flank pain. Oral intake was resumed on postoperative day 1, and ambulation was encouraged. There were no episodes of fever, hematuria, or lower urinary tract symptoms during the hospital course. Postoperative renal function remained stable, with serum creatinine, urea, sodium, potassium, and uric acid levels within normal limits. The patient was discharged in stable condition on postoperative day 2 with appropriate analgesics, antibiotics, and advice for follow-up. The ureteral stent was removed after 4 weeks during outpatient follow-up.

## Discussion

Ureteroscopy is widely regarded as a safe and effective intervention for ureteral and renal calculi; however, procedural difficulty can arise when stone disease is accompanied by anatomical distortion or altered urinary tract orientation. Conditions associated with hydronephrosis and ureteral obstruction are known to increase technical complexity by affecting ureteral compliance, luminal diameter, and scope maneuverability. Keenan RA et al. emphasized that symptomatic hydronephrosis, irrespective of etiology, alters ureteral dynamics and necessitates careful procedural planning to minimize intraoperative complications [6].

Anatomical abnormalities of the ureter represent an important yet often underrecognized cause of difficult ureteroscopy. Aberrant ureteral course, compression, or displacement can significantly interfere with endoscopic access and instrument navigation. Sharma PK et al. highlighted how rare anatomical variants such as retrocaval ureter can present with hydroureteronephrosis and create substantial challenges during endoscopic management [7]. Similarly, congenital or acquired anatomical distortions may result in acute angulation and nonlinear ureteral trajectories, as observed in the present case, increasing resistance during scope advancement and stone manipulation.

Complex urinary tract anatomy may also coexist with stone disease, further compounding procedural difficulty. Mahesvara IB et al. reported that duplex collecting systems encountered in the management of ureteral calculi can alter normal drainage patterns and complicate endourological access, underscoring the importance of recognizing anatomical variations prior to intervention [8]. In the present case, spinal deformity and pelvic distortion contributed to abnormal ureteral alignment, which was visually corroborated by post-procedure stent angulation, reinforcing the impact of extrinsic anatomy on ureteroscopic ergonomics.

While ureteroscopy remains a preferred modality for stone management across age groups, outcomes are influenced by patient-specific and anatomical factors. Ureteroscopy remains a preferred modality for the management of ureteral calculi, with high stone-clearance rates and acceptable morbidity profiles. A systematic review and meta-analysis of randomized controlled trials on the treatment of ureteral and renal stones demonstrated that ureteroscopy provides effective stone clearance across a range of ureteral and renal calculi, while emphasizing that outcomes are influenced by stone characteristics and patient-specific anatomical factors [9]. In anatomically complex cases, such as the present one, procedural success depends not only on the choice of modality but also on careful preoperative evaluation and intraoperative adaptability. Current guideline-based management of ureteric calculi emphasizes that treatment selection should be individualized according to stone size, location, density, patient anatomy, and anticipated technical difficulty. In complex anatomical situations, preoperative imaging and intraoperative adaptability are critical to minimize ureteral trauma and optimize stone clearance [10].

Flexible ureteroscopy may be a useful alternative or adjunct in selected complex cases, particularly when semirigid access is unsafe or unsuccessful because of abnormal ureteral angulation or difficult upper tract access. In the present case, however, the stone was located in the distal ureter and was ultimately managed successfully with cautious semirigid ureteroscopy and combined lithotripsy. Thus, flexible ureteroscopy remained a backup strategy rather than the primary modality.

Stone-related factors also contribute significantly to procedural difficulty. Impacted calculi have been linked to chronic inflammatory changes in the ureteral wall, leading to reduced elasticity and increased resistance to instrumentation. Abdrabuh AM et al. demonstrated that increased preoperative ureteral wall thickness is predictive of stone impaction and correlates with increased technical difficulty during laser ureteroscopic lithotripsy [11]. In the present case, the combination of stone impaction, mucosal edema, and granulation tissue resulted in a narrowed distal ureter, necessitating careful torque control, combined energy fragmentation, and prolonged instrumentation.

Risk stratification tools and scoring systems have been proposed to predict outcomes and guide procedural planning in complex stone disease. He Q et al. highlighted the value of nephrolithometry-based scoring systems in predicting outcomes for both percutaneous nephrolithotomy and flexible ureteroscopy, reinforcing the importance of preoperative assessment in anticipating procedural difficulty [12]. Although nephrolithometry-based scoring systems are useful for anticipating procedural complexity in stone disease, they were not formally applied in the present case because this was an individualized, case-based management decision rather than a protocolized comparative analysis. Nonetheless, the preoperative CT findings, namely stone density, distal impaction, upstream dilatation, and distorted anatomy, served as practical indicators of likely procedural difficulty.

This case demonstrates that flexible ureteroscopy requires detailed understanding of stone characteristics, anatomical distortion, and intraoperative adaptability. Reliance on multiple fragmentation modalities, together with meticulous endoscopic technique and delayed ureteral stent placement, enabled safe and effective stone clearance even in the presence of unfavorable anatomy. Such challenges and critical intraoperative decisions must be recognized and addressed appropriately to optimize outcomes and minimize complications during complex ureteroscopic procedures.

Limitations

This report describes a single technically challenging case; therefore, the observations cannot be generalized to all patients undergoing ureteroscopy in complex anatomical settings. Formal ureteral edema grading and nephrolithometry-based scoring were not applied in this case, and flexible ureteroscopy, although considered as an alternative option, was not ultimately required. In addition, the report is limited by the absence of intraoperative endoscopic images. Nevertheless, the case remains clinically valuable because it illustrates how preoperative imaging, anatomical distortion, skeletal comorbidity, and adaptive intraoperative decision-making can influence successful endourological management.

## Conclusions

Difficult ureteroscopy can occur when stone disease is compounded by altered anatomical orientation and chronic inflammatory changes of the ureter. This case highlights how spinal deformity, distorted pelvic anatomy, and an impacted high-density distal ureteric calculus can significantly impose technical challenges during ureteroscopic access and stone fragmentation. Careful intraoperative assessment, controlled endoscopic manipulation, and strategic use of complementary lithotripsy modalities were essential in achieving successful stone clearance while minimizing the risk of ureteral injury.

Recognition of anatomical distortion on preoperative imaging, anticipation of procedural difficulty, and readiness to modify operative strategy are critical for safe and effective ureteroscopic management in complex cases. This report underscores the importance of individualized surgical planning and adaptive endourological decision-making when managing challenging ureteroscopic scenarios.
